# Breast Cancer Cell Line-Specific Responses to Insulin: Effects on Proliferation and Migration

**DOI:** 10.3390/ijms26157523

**Published:** 2025-08-04

**Authors:** Mattia Melloni, Domenico Sergi, Angelina Passaro, Luca Maria Neri

**Affiliations:** 1 Department of Translational Medicine, University of Ferrara, Via Luigi Borsari 46, 44121 Ferrara, Italy; mattia.melloni@unife.it (M.M.); domenico.sergi@unife.it (D.S.); 2 Laboratory for Technologies of Advanced Therapies (LTTA)—Electron Microscopy Center, University of Ferrara, Via Luigi Borsari 46, 44121 Ferrara, Italy

**Keywords:** type 2 diabetes mellitus, hyperinsulinemia, hyperglycaemia, breast cancer, insulin signalling

## Abstract

Breast cancer (BC) progression appears to be significantly influenced by the diabetic microenvironment, characterised by hyperglycaemia and hyperinsulinemia, though the exact cellular mechanisms remain partly unclear. This study investigated the effects of exposure to supra-physiological levels of glucose and insulin on two distinct BC cell models: hormone-responsive MCF-7 cells and triple-negative MDA-MB-231 cells. To evaluate the effects triggered by high insulin level in different BC cell subtypes, we analysed the activation status of PI3K/AKT and MAPK pathways, cell proliferation, cell distribution in cell cycle phases and cell migration. High insulin level significantly activates the insulin metabolic pathway via AKT phosphorylation in both cell lines while inducing pro-proliferative stimulus and modulation of cell distribution in cell cycle phases only in the hormone-responsive MCF-7 cell line. On the contrary, high-glucose containing medium alone did not modulate proliferation nor further increased it when combined with high insulin level in both the investigated cell lines. However, following insulin treatment, the MAPK pathway remained unaffected, suggesting that the proliferation effects in the MCF-7 cell line are mediated by AKT activation. This linkage was also demonstrated by AKT phosphorylation blockade, driven by the AKT inhibitor MK-2206, which negated the proliferative stimulus. Interestingly, while MDA-MB-231 cells, following chronic hyperinsulinemia exposure, did not exhibit enhanced proliferation, they displayed a marked increase in migratory behaviour. These findings suggest that chronic hyperinsulinemia, but not hyperglycaemia, exerts subtype-specific effects in BC, highlighting the potential of targeting insulin pathways for therapeutic intervention.

## 1. Introduction

Diabetes is a metabolic disorder characterised by chronic hyperglycaemia driven by an absolute or relative insulin deficiency [1]. The incidence of diabetes is increasing worldwide, affecting 537 million people in 2021, and it is expected to affect 783 million by 2045 [2]. Remarkably, 95% of people affected by diabetes are suffering from type 2 diabetes mellitus (T2DM) [3]. The exponential increase in T2DM is driven by obesity, which is key in promoting insulin resistance (IR), the main hallmark of T2DM [4]. Indeed, the onset of full-blown T2DM is preceded by IR, referred to as a blunted response of insulin tissue target to the metabolic effects of this hormone [5]. In response to IR, the pancreatic β-cells orchestrate a compensatory insulin hyper-secretory response, thereby leading to hyperinsulinemia [6]. However, hyperinsulinemia is not merely a hallmark of prediabetes but also a driver of multiple metabolic and cellular dysregulations [7,8].

In line with this, growing evidence indicates that chronic hyperinsulinemia may contribute to an increased risk of cancer [9,10,11], with epidemiological studies supporting a pathophysiological link between hyperinsulinemia and the world’s most common type of cancer, breast cancer (BC) [8,12,13,14]. Moreover, hyperinsulinemia has been related to the initiation, progression, and spread of BC, potentially disrupting the regulation of cell adhesion molecules and facilitating the epithelial-to-mesenchymal transition, a key process driving cancer cell migration [15,16].

In addition to hyperinsulinemia, chronic hyperglycaemia represents a significant metabolic alteration in T2DM related to increased BC risk [17] and is responsible for profound cellular and molecular consequences [18]. Indeed, persistent hyperglycaemia induces oxidative stress and promotes chronic low-grade inflammation [19]. Furthermore, hyperglycaemia triggers the activation of oncogenic pathways [17], as an instance via the Warburg effect responsible for increasing aerobic glycolysis-dependent pathways, which lead to lactate production, microenvironment acidification, and cancer promotion [20].

Consistent with epidemiological data, in vitro experimental evidence suggests that hyperinsulinemia can promote the proliferation and migration of BC cells [21] while, on the contrary, other evidence states that the pro-proliferative effect of this hormone is limited to breast epithelial cells [22]. Meanwhile, in vitro studies examining the link between hyperglycaemia and BC are still scarce, and many have employed glucose concentrations that differ from those observed in human pathophysiology [23,24,25].

At the molecular level, the binding of insulin to its cognate receptor activates downstream signalling pathways, namely the phosphoinositide-3-kinase (PI3K)/protein kinase B (AKT) and mitogen-activated protein kinase (MAPK), which, in turn, regulate cell proliferation and survival [26]. Although hyperinsulinemia in the literature has been widely proposed as a driver of BC promotion and dissemination [7,8,16,27,28], studies elucidating its involvement in PI3K and MAPK pathways are still scarce [29,30,31,32,33].

Thus, the aim of this study was to evaluate the effects of chronic hyperinsulinemia and hyperglycaemia in MDA-MB-231 and MCF-7 BC cell proliferation. In addition, the hyperinsulinemia-driven modulation of PI3K and MAPK pathways and its impact on BC cell migration were evaluated.

## 2. Results

### 2.1. Breast Cancer Cells Respond to Acute Insulin Challenge

**Figure 1:**
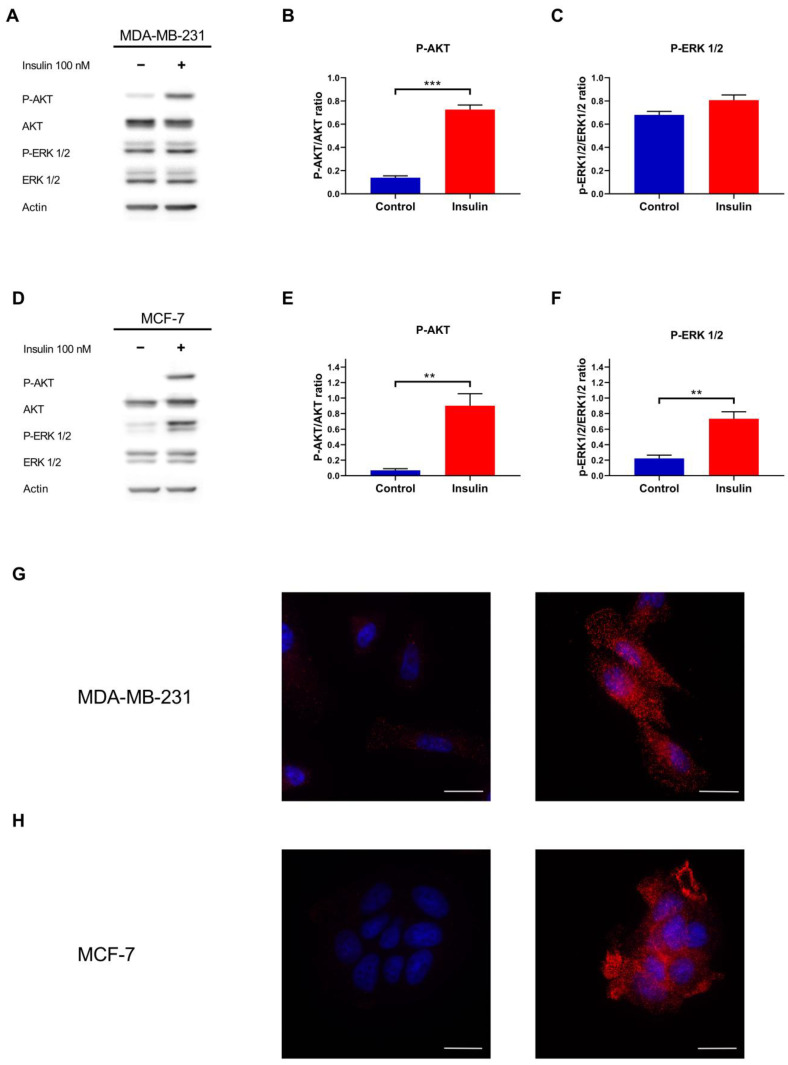
Insulin metabolic and mitogenic pathways analysis in MDA-MB-231 and MCF-7 cell lines. (**A**) Representative Western blot of the phosphorylation of AKT and ERK1/2 proteins in MDA-MB-231 cells treated with or without insulin 100 nM. Densitometric analysis of the phosphorylation of AKT (**B**) and ERK1/2 (**C**) in MDA-MB-231, normalised for their unphosphorylated form. (**D**) Representative Western blot of AKT and ERK1/2 phosphorylation after MCF-7 treatment with or without insulin. Densitometric analysis of the phosphorylation of AKT (**E**) and ERK1/2 (**F**) in MCF-7, normalised for their unphosphorylated form. (**G**) Immunofluorescence analysis of the phosphorylation of AKT Ser473 in untreated (**left**) and insulin-treated MDA-MB-231 cells (**right**). (**H**) Immunofluorescence analysis of the phosphorylation of AKT Ser473 (red) in untreated (**left**) and insulin-treated MCF-7 cells (**right**). Nuclei were counterstained with DAPI (blue). Scale bar = 25 µm. Western blot data are the mean of three independent biological replicates ± SEM. Statistical analysis was performed using a two-tailed *t*-test. ** *p* < 0.01, *** *p* < 0.001.

To assess whether MDA-MB-231 and MCF-7 BC cells may respond to insulin, they were exposed to 100 nM of this anabolic hormone for 15 min. Insulin induced AKT phosphorylation on Ser473 in MDA-MB-231 (Figure 1A,B) and MCF-7 (Figure 1D,E) cell lines (*p* < 0.001 and *p* = 0.006, respectively). On the contrary, insulin induces the phosphorylation of ERK1/2 on Thr202/Tyr204 residues in MCF-7 (*p* = 0.007) but not in MDA-MB-231 (*p* = 0.097) (Figure 1D,F).

AKT phosphorylation was corroborated by immunofluorescence, with insulin-increasing cytoplasmic and membrane staining in MDA-MB-231 (Figure 1G) and MCF-7 (Figure 1H) cell lines.

Taken together, these results indicate that insulin activates the PI3K/AKT signalling axis in both triple-negative and luminal breast cancer subtypes, whereas ERK1/2 pathway activation is restricted to the hormone receptor positive MCF-7, reflecting a subtype specific divergence in downstream insulin signalling.

### 2.2. High Insulin Level Only Induces Cell Proliferation in MCF-7 but Not in MDA-MB-231 Cell Lines

**Figure 2:**
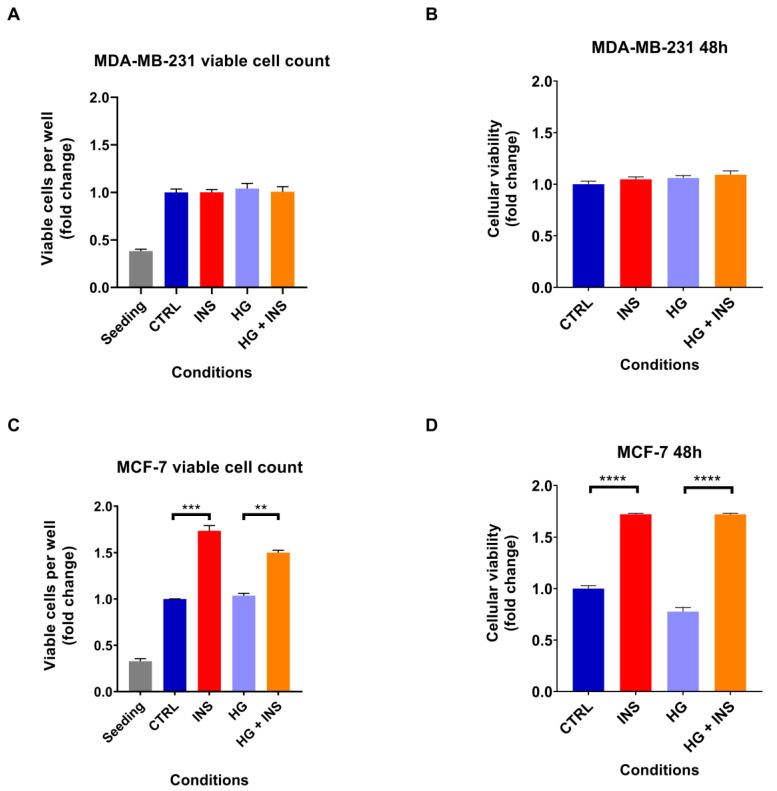
Chronic insulin effect on cell proliferation after 48 h of treatment with 25 nM insulin in the presence or absence of high-glucose containing medium. MDA-MB-231 cell proliferation analysed by flow cytometry (**A**) and by MTT assay (**B**). MCF-7 cell proliferation investigated by flow cytometry (**C**) and by MTT assay (**D**). Data are expressed in fold change relative to the control condition. Data are reported as the mean of three independent biological replicates ± SEM, except for the cell viability assay on MDA-MB-231 cells where the replicates were five and for the MTT assay on MCF-7 cells where the replicates were four. Statistical analysis was performed using the one-way Anova test. ** *p* < 0.01, *** *p* < 0.001, **** *p* < 0.0001. CTRL, control; INS, 25 nM insulin; HG, hyperglycaemia.

In light of the well-recognised mitogenic effect of insulin [30,34] and the increased glucose consumption by cancer cells [35], it was investigated whether high insulin level or high-glucose containing medium alone, or in combination, were able to promote the proliferation of both MDA-MB-231 and MCF-7 cell lines. A 48 h treatment with 25 nM insulin alone did not enhance the proliferation of MDA-MB-231 cells (*p* > 0.999) (Figure 2A), whereas it induced MCF-7 cell growth by 40% (*p* < 0.001) (Figure 2C), as also confirmed by MTT assays (*p* = 0.658 and *p* < 0.001, respectively) (Figure 2B,D). Instead, 48 h treatment with high-glucose containing medium did not increase cell proliferation in either cell line (*p* = 0.917 and *p* = 0.706, for MDA-MB-231 and MCF-7 viable cell count, respectively) (Figure 2A,C) as also confirmed by MTT assays (*p* = 0.476 for MDA-MB-231 and *p* = 0.001 for MCF-7) (Figure 2B,D). In addition, high-glucose containing medium did not enhance the pro-proliferative effect of insulin in MCF-7 cells as shown by viable cell count (*p* > 0.999), although the MTT assay revealed a reduction in metabolic activity in cells exposed to high glucose levels (*p* = 0.034). This result may reflect an alteration in mitochondrial function induced by high glucose levels, rather than a decrease in cell viability (Figure 2C,D).

Given the inability of high-glucose containing medium to promote cell proliferation, we decided not to investigate this condition further and instead focus exclusively on high insulin level.

### 2.3. Breast Cancer Cells Respond to Chronic Insulin Stimuli

**Figure 3:**
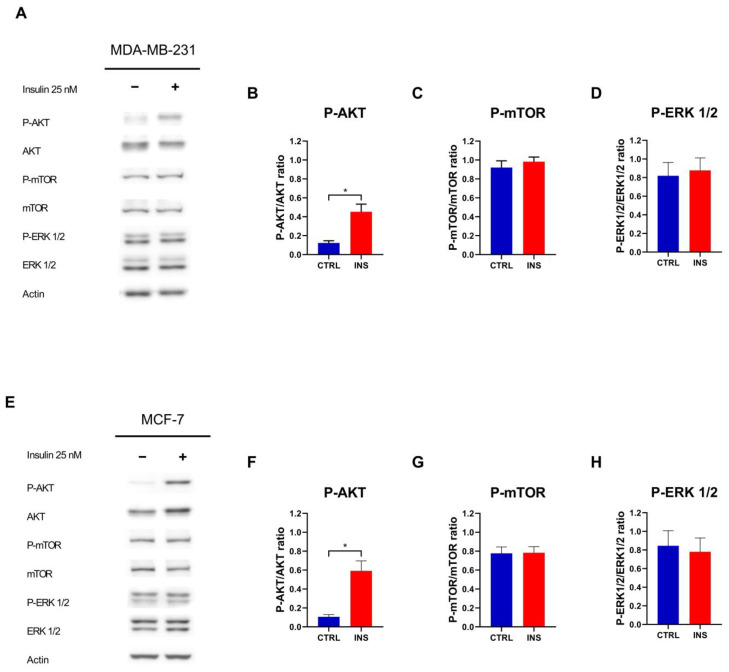
Insulin pathway activation following chronic insulin treatment. (**A**) Representative Western blot of the phosphorylation of AKT, mTOR, and ERK 1/2 in MDA-MB-231 cells treated with or without insulin 25 nM for 48 h. Densitometric analysis of the phosphorylation of AKT (**B**), mTOR (**C**), and ERK 1/2 (**D**) in MDA-MB-231 cells, normalised for their unphosphorylated form. (**E**) Representative Western blot of the phosphorylation of AKT, mTOR, and ERK 1/2 in MCF-7 cells treated with or without insulin 25 nM for 48 h. Densitometric analysis of the phosphorylation of AKT (**F**), mTOR (**G**), and ERK 1/2 (**H**) in MCF-7 cells, normalised for their unphosphorylated form. Western blot data are reported as the mean of three independent biological ± SEM. Statistical analysis was performed using a two-tailed *t*-test. * *p* < 0.05. CTRL, control; INS, 25 nM insulin.

Given the absence of proliferation induction triggered by high-glucose containing medium, it was decided not to further investigate this condition and instead focus on high insulin level. Accordingly, to determine whether the effects of insulin are mediated by alterations in insulin signalling, the phosphorylation of proteins downstream of the insulin receptor was examined following 48 h of exposure to this anabolic hormone. Insulin induced AKT phosphorylation in both MDA-MB-231 (*p* = 0.017) (Figure 3A,B) and MCF-7 (*p* = 0.010) (Figure 3E,F) cells but failed to induce the phosphorylation of mTOR (*p* = 0.514 and *p* = 0.945 for MDA-MB-231 and MCF-7, respectively) and ERK 1/2 proteins (*p* = 0.781 and *p* = 0.785 for MDA-MB-231 and MCF-7, respectively) (Figure 3C,D,G,H).

### 2.4. High Insulin Level Modulates Cell Cycle in MCF-7 but Not in MDA-MB-231 Cell Lines

**Figure 4:**
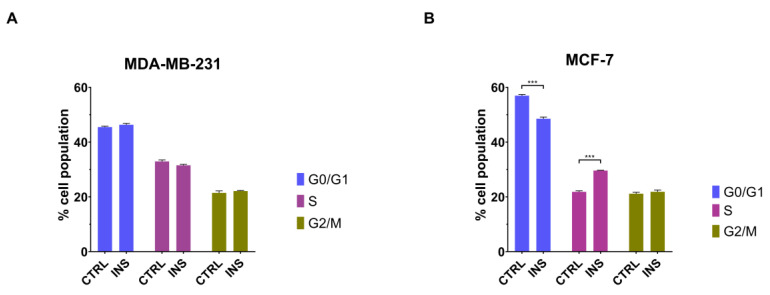
Chronic insulin effect on cell cycle. Cell cycle analysis of MDA-MB-231 cells (**A**) and MCF-7 (**B**) after 48 h of exposure to 25 nM insulin. Data are reported as the means of three independent biological replicates ± SEM. Statistical analysis was performed using a two-way Anova followed by Sidak’s post hoc test. *** *p* < 0.001. CTRL, control; INS, 25 nM insulin.

Considering the ability of inulin to induce the proliferation of MCF-7 but not MDA-MB-231 cell line, it was next evaluated whether insulin could affect the cell cycle in these cells. A 48 h treatment with 25 nM insulin did not influence the cell cycle of MDA-MB-231 cells (*p* = 0.541, *p* = 0.142 and *p* = 0.699 for G0/G1, S, and G2/M phases, respectively) (Figure 4A). On the contrary, high insulin level increased the percentage of MCF-7 cells in the S phase (*p* < 0.001) while decreasing the number of cells in the G0/G1 phases (*p* < 0.001) of the cell cycle (Figure 4B). However, these effects occurred without changes in the percentage of cells on the G2/M phase of the cell cycle (*p* = 0.689) (Figure 4B).

### 2.5. High Insulin Level-Induced Proliferation of MCF-7 Cells Is AKT-Dependent

**Figure 5:**
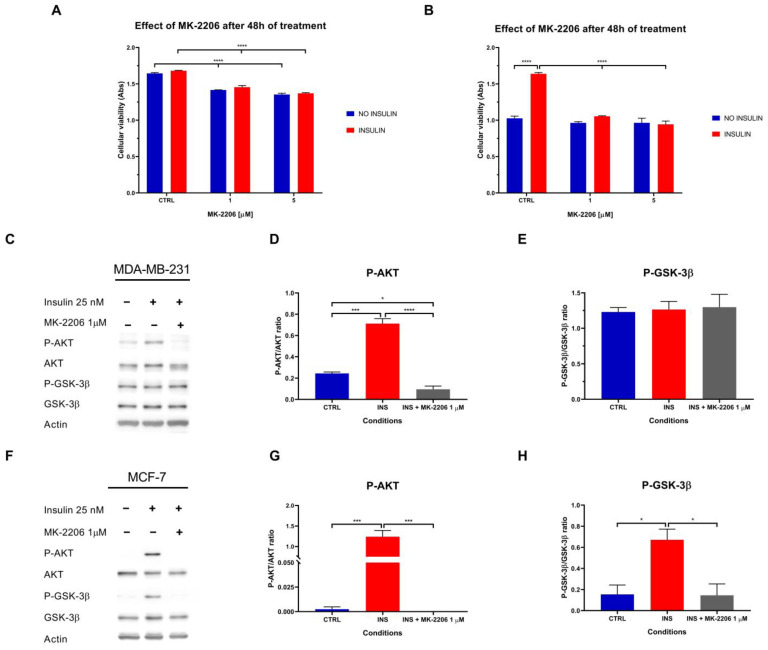
Effect of AKT activation blockade in cell viability and metabolic insulin pathway. MK-2206-driven AKT blockade effect on the viability of MDA-MB-231 (**A**) and MCF-7 (**B**) cells treated for 48 h with or without high insulin levels. (**C**) Representative Western blot of the phosphorylation of the proteins AKT and GSK-3β in MDA-MB-231 cells treated with or without insulin 25 nM and MK-2206 for 48 h. Densitometric analysis of the phosphorylation of AKT (**D**) and GSK-3β (**E**) in MDA-MB-231 cells, normalised for their unphosphorylated form. (**F**) Representative Western blot of the phosphorylation of the proteins AKT and GSK-3β in MCF-7 cells treated with or without insulin 25 nM and MK-2206 for 48 h. Densitometric analysis of the phosphorylation of AKT (**G**) and GSK-3β (**H**) in MCF-7 cells, normalised for their unphosphorylated form. Reported data are reported as the means of three independent biological replicates ± SEM, except for the control condition of the MTT assay with MCF-7 cells where the replicates are six. Statistical analysis was performed using a two-way Anova test followed by Tukey’s post hoc test for proliferation data comparison and using a one-way Anova test followed by Tukey’s post hoc test for Western blot analysis. * *p* < 0.05, *** *p* < 0.001, **** *p* < 0.0001.

To clarify the mechanism underlying insulin-induced cell proliferation, we investigated whether this effect depended on AKT by inhibiting its phosphorylation with 1 and 5 µM MK-2206. In MDA-MB-231, the AKT inhibitor decreased cell proliferation independently of the presence of insulin (*p* < 0.001 in both conditions), which, as previously demonstrated, is ineffective in further increasing cell proliferation (Figure 5A).

Instead, in agreement with the fact that insulin promoted the proliferation of MCF-7 cells, the AKT inhibitor successfully negated insulin-dependent cell proliferation (*p* = 0.270 for MK-2206 1 µM and *p* = 0.967 for MK-2206 5 µM) (Figure 5B). Treatment with 1 µM MK-2206 for 48 h reduced the number of viable MDA-MB-231 cells by 15% under insulin-treated and untreated conditions. Higher concentrations of MK-2206 did not further affect cell viability (Figure 5B).

Considering the ability of MK-2206 to inhibit cell proliferation, the effect of insulin signalling transduction was evaluated. As expected, MK-2206 effectively abolished insulin-induced AKT phosphorylation in MDA-MB-231 (Figure 5C,D) and MCF-7 (Figure 5F,G), whereas reduced GSK-3β phosphorylation only in MCF-7 (Figure 5F) as evidenced by the densitometric analysis (*p* = 0.022) (Figure 5H).

### 2.6. High Insulin Level Induces Pro-Migration Phenotype in MDA-MB-231 but Not in MCF-7

**Figure 6:**
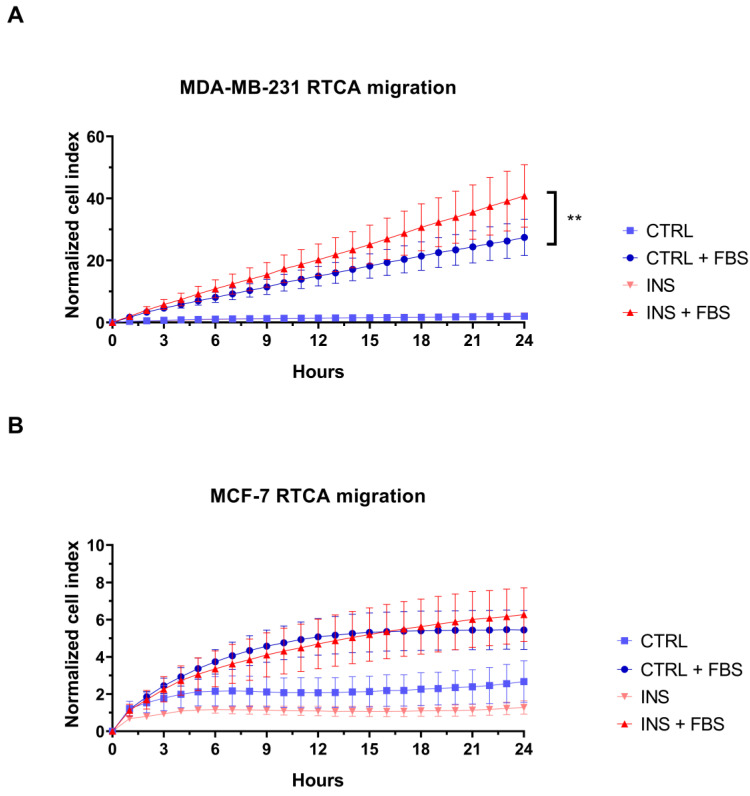
The effect of high insulin level on breast cancer cell migration. Real-time migration curves of MDA-MB-231 (**A**) and MCF-7 (**B**) cells treated with or without insulin 25 nM and in the presence or not of the chemoattractant. In panel A, the hyperinsulinemia condition curve is not visible due to complete overlap with the control curve. RTCA curves are the means of at least three technical replicates ± SEM. Statistical analysis was performed using a linear regression test. ** *p* < 0.01. RTCA, Real-Time Cell Analysis; CTRL, control; FBS, 10% Foetal Bovine Serum; INS, insulin.

In light of the previously reported ability of insulin to promote primary tumour growth and metastasis [28] and the higher breast cancer mortality in subjects with high insulin level [7], we evaluated if this condition could enhance the migration of the studied BC cell lines. For this purpose, the RTCA-based migration assay was performed. In the presence of the chemoattractant, 48 h insulin treatment induced a higher migration rate when compared with control MDA-MB-231 cells (*p* = 0.001) (Figure 6A). In contrast to MDA-MB-231, MCF-7 cells did not show a similar migration ability since the addition of insulin did not increase the migration rate of chemoattracted cells (*p* = 0.299) (Figure 6B).

## 3. Discussion

This study investigated the impact of hyperinsulinemia and high-glucose on BC cell proliferation, with particular attention to the modulation of cell signalling, proliferation, and invasiveness induced by insulin. Our findings demonstrate that both the cell lines are responsive to insulin as indicated by the activation of the PI3K/AKT signalling pathway following insulin stimulation. This characteristic made them suitable models to investigate the impact of hyperinsulinemia on BC pathogenesis.

In line with our in vitro observations, clinical data likewise implicate hyperinsulinemia in fostering PI3K/AKT signalling within human BC. Immunohistochemical studies on specimens from patients exhibiting elevated insulin levels consistently show increased AKT phosphorylation across multiple BC molecular subtypes, indicating that hyperinsulinemia is associated with enhanced AKT activation in situ [36]. These findings suggest the effects of insulin extend beyond systemic metabolic regulation with this hormone exerting an oncogenic stimulation in the tumour microenvironment. Furthermore, patients with higher circulating insulin levels display poorer outcomes and shorter survival [37], a pattern that may reflect chronic engagement of the PI3K/AKT axis driving proliferation and resistance to apoptosis [38].

Conversely, the protein ERK 1/2, the last substrate in the MAPK pathway, was activated only in MCF-7 cells following acute insulin exposure, while such activation was not observed in MDA-MB-231 cells. This discrepancy likely reflects the distinct characteristics of these cell lines. Specifically, MDA-MB-231 cells, which have a higher proliferation rate independent of insulin, also exhibit elevated basal ERK 1/2 activation. Moreover, previous reports suggest that the ability of insulin to stimulate the mitogenic MAPK pathway in MCF-7 cells may be linked to ER expression, which enhances the insulin-mediated mitogenic effects by upregulating insulin receptor substrate 1 and further promoting MAPK signalling [31].

Interestingly, proliferation assays revealed that a 48 h insulin challenge significantly enhanced the growth of only MCF-7 cells, with a 40% increase in viable cell count, whereas the proliferation of MDA-MB-231 was not modulated. In line with our results, the differential response of the studied BC cell line to chronic high insulin level has also been reported by other authors [32,39]. The distinct responses of BC cells to high insulin level can be attributed to their intrinsic characteristics. Indeed, this subtype-related proliferative response underlines why patients with elevated plasma insulin levels and hormone receptor-positive BC often exhibit worse 5-year survival rates, despite endocrine therapy [40].

Furthermore, as previously documented, ER-positive breast tumours frequently exhibit elevated levels of insulin-like growth factor-1 receptor (IGF-1R) and its downstream adaptor protein insulin receptor substrate-1 (IRS-1). In fact, hyperinsulinemia not only directly engages insulin and IGF-1R but also amplifies mitogenic signalling by boosting free IGF-1 availability, which activates IGF-1R, initiating signalling cascades such as PI3K, inhibiting apoptosis, and promoting protein synthesis [26]. Such interplay between insulin, IGF-1, and their receptors underscore the critical role of metabolic dysregulation in BC promotion.

In addition, in vitro studies have shown that activation of the IGF-1R/IRS-1 signalling axis in ER-positive cells can promote their proliferation. Conversely, ER-negative breast cancer cells typically express lower levels of IGF-1R and do not exhibit a mitogenic response to IGF-1 stimulation [41].

However, raising the glucose concentration from 5,5 mM (100 mg/dL, physiological) to 13 mM (234 mg/dL, hyperglycaemia) failed to promote cell proliferation. One plausible explanation is that the basal metabolism of MCF-7 cells is already optimised at 5,5 mM glucose, with no further metabolic flux enhancement upon additional glucose supplementation. The overall observation suggests that the mitogenic stimulus provided by insulin can be substantially more potent than the adjunct availability of glucose. Moreover, the limitations inherent in the in vitro model must be considered, as these conditions may not fully recapitulate the nutrient supply and glucose consumption encountered in vivo. These findings indicate that, despite the intuitive assumption that higher glucose availability should enhance proliferation, BC cells respond more strongly to the direct mitogenic effects of insulin.

In keeping with the investigation of the effect on the proliferation of BC cells, we have next evaluated the impact of this hormone on the cell cycle. In accordance with the fact that MDA-MB-231 did not show an increase in their proliferation rate in response to high insulin level, this hormone did not affect the cell disposition in G0/G1, S, and G2/M cell cycle phases. Conversely, insulin-responsive MCF-7 showed an increase in cellular disposition in the G0/G1 and S phases of the cell cycle after 48 h of treatment. This can be explained by the insulin-mediated increase in cyclin D1 expression, even in the absence of oestrogen, which mediates the entrance of resting cells in the cell cycle and progress through the G1–S transition, as already reported by other authors [42]. This dependency on cyclin D1-driven checkpoint control highlights the cyclin D1-CDK4/6 axes as an interesting therapeutic target [43] that warrants further preclinical and clinical investigation in ER-positive BC patients with hyperinsulinemia. However, these changes in the cell cycle, surprisingly, did not lead to an increase in the number of cells in the G2/M phase of the cell cycle. This phenomenon was previously reported in different cell lines in which insulin had no significant impact on the G2/M phase distribution. However, this hormone dose-dependently reduced the percentage of cells in the G1 phase with an increased S phase population, concluding that this is a phenomenon generally observed when the G1-S cell cycle transition is accelerated [44].

Under chronic insulin stimulation, both BC cell models demonstrated persistent activation of AKT, whereas its downstream target mTOR and the MAPK-related protein ERK 1/2 remained unresponsive. Despite the lack of further mTOR activation following chronic insulin stimulus, our findings indicate that both MCF-7 and MDA-MB-231 breast cancer cell lines exhibit a constitutively active mTOR pathway at baseline, a feature that is documented by other works [45,46]. This inherent hyperactivation likely indicates that chronic insulin exposure does not further enhance mTOR phosphorylation. Despite insulin’s ability to activate AKT, the lack of additional mTOR phosphorylation suggests that the regulatory circuitry responsible for mTOR activation might already be operating at its peak or be controlled by intrinsic negative feedback loops.

It is plausible that once mTOR is maximally engaged under basal conditions, further stimulation fails to produce a measurable increase in its phosphorylation status. Alternatively, the activation of AKT following insulin treatment could initiate feedback mechanisms that dampen further mTOR activation, thereby maintaining a stable signalling environment.

Adding to this, the lack of ERK 1/2 activation suggests that prolonged insulin exposure may induce selective desensitisation of the MAPK pathway or trigger feedback inhibitory mechanisms that preserve metabolic signalling over mitogenic responses. Although studies evaluating ERK 1/2 activation under prolonged insulin exposure are limited, the observed results in MDA-MB-231 align with previous findings where 24 h chronic insulin exposure failed to induce ERK 1/2 activation [24]. Therefore, it appears that chronic insulin treatment activates AKT signalling in both MDA-MB-231 and MCF-7 cells, whereas ERK signalling becomes desensitised in MCF-7 cells under prolonged insulin exposure.

To assess the AKT-dependent pro-proliferative effect triggered by insulin, both MDA-MB-231 and MCF-7 cells were treated with the AKT inhibitor MK-2206. Our findings suggested that the proliferation of MDA-MB-231 is dependent on the phosphorylation status of AKT, with MK-2206 inducing a similar decrease in cell proliferation in cells treated with or without insulin. In accordance with this, Western blot analysis of MDA-MB-231 cells treated with insulin and MK-2206 showed the complete inhibition of AKT activation, while the activation of GSK-3β remained unaffected. The sustained activity of GSK-3β, despite the complete dephosphorylation of AKT, may be explained by the involvement of alternative regulatory mechanisms. Indeed, while AKT phosphorylates GSK-3β on Ser9, which inhibits its kinase activity [47], other signalling pathways can also regulate GSK-3β. These include kinases such as p70 S6 kinase, protein kinase A, and MAPK [48,49], which have been shown to phosphorylate and modulate GSK-3β activity. These results indicate that, in MDA-MB-231 cells, alternative pathways independent of AKT predominantly regulate GSK-3β activity. This suggests that GSK-3β may not be solely regulated by AKT in this specific cellular context, highlighting the complexity of cellular signalling networks.

In keeping with the AKT inhibitor MK-2206, the low impact observed in MDA-MB-231 cell proliferation after AKT signalling blockade may be due to this cell line’s inherently high basal proliferation rate and reliance on alternative growth-regulatory proteins, such as hypoxia inducible factor-1 [50]. Indeed, HIF-1 has been related to act as a vascularisation stimulator inducing the expression of vascular endothelial growth factor [51]; shift cellular metabolism toward anaerobic glycolysis, thereby ensuring a rapid supply of ATP and metabolic intermediates required for biomolecules synthesis in proliferating cells [52]; and interact with pathways such as PI3K/AKT, thereby amplifying pro-proliferative and anti-apoptotic signals [53]. On the contrary, in MCF-7 cells, the proliferative effect induced by insulin was negated by MK-2206, suggesting the cell growth of these cells is AKT-dependent. Nevertheless, the basal proliferation of MCF-7 cells not treated with insulin may be independent of AKT activation as it was not affected by this AKT inhibitor. Western blot analysis of MCF-7 cells treated with insulin and MK-2206 showed complete inhibition of AKT phosphorylation and a strong phosphorylation reduction in the AKT downstream protein GSK-3β, further supporting the strong relationship between the insulin metabolic pathway, the cell cycle modulation, and the proliferation of this cell line.

Real-time migration analysis showed that chronic insulin exposure induced a pro-migratory phenotype in MDA-MB-231 cells. In agreement with these findings, the increase in MDA-MB-231 cell migration has been related to insulin-induced downregulation of E-cadherin expression, accompanied by an increase in N-cadherin and vimentin expression, which are involved in Epithelial to Mesenchymal Transition (EMT). In addition, the insulin-induced nuclear receptor subfamily 2, group F, member 2, gene overexpression has been related to the promotion of EMT and, as a consequence, to an increase in BC cell migration potential [16]. Further supporting our findings, in vivo studies on high insulin level-treated mouse showed that this hormone enhances BC to lung metastasis. This effect is mediated by the upregulation of the transcription factor cellular Myc, which is associated with increased levels of matrix metalloproteinase-9 and vascular endothelial growth factor, both key drivers of metastatic progression [54]. Thus, despite insulin’s failure to increase the proliferation of these cells, it increased their migration potential. In contrast, insulin did not increase the already low basal migration potential of MCF-7 cells.

These findings imply that hyperinsulinemia may contribute significantly to metastatic dissemination in more aggressive, triple-negative BC cells.

## 4. Materials and Methods

### 4.1. Cell Culture

The human BC cell line MDA-MB-231 (negative for oestrogen receptor (ER), progesterone receptor (PR), and human epidermal growth factor receptor 2 (HER2)) was obtained from LGC Standards (Middlesex, UK), while the cell line MCF-7 (positive for ER and PR but negative for HER2) [55] was purchased from the American Type Culture Collection (Rockville, MD, USA). Both the cell lines were cultured in Dulbecco′s Modified Eagle′s Medium (DMEM)-Low Glucose (5.5 mM glucose) (Euroclone, Pero, MI, Italy) supplemented with 100 U/mL Penicillin-Streptomycin, both purchased from Sigma-Aldrich (St. Louis, MO, USA), and 10% Foetal Bovine Serum (FBS) obtained from Microgen (Microgem, Naples, NA, Italy) and incubated at 5% CO_2_ and 90% relative humidity at 37 °C.

### 4.2. Cell Treatment

After an overnight cell adhesion, cells were treated for 48 h with either DMEM-LG or DMEM-High Glucose (HG) (13 mM glucose), supplemented with 2% FBS and in the presence or absence of 25 nM insulin [25,56,57] (Sigma-Aldrich). To assess the responsiveness of both cell lines with insulin, cells were challenged for 15 min with 100 nM insulin [58]. Cells were grown in either 6-, 16- or 96-well plates, depending on the assay to be performed.

Furthermore, both cell lines were treated with the AKT inhibitor MK-2206 (Selleck Chemicals LLC, Houston, TX, USA) at 1 and 5 µM [59,60] in the presence or absence of 25 nM of human insulin.

### 4.3. Western Blot Analysis of Protein Phosphorylation

Western blotting assays were conducted to evaluate the activation of the mitogen and metabolic insulin pathway following acute (100 nM) or chronic (25 nM) insulin treatment.

After treatments, cells were lysed using RIPA buffer supplemented with 1% v/v phosphatase and protease inhibitors (Sigma-Aldrich). Lysates were kept on ice for 30 min and then centrifuged for 10 min at 4 °C at 13,000× *g* to remove cellular debris. Protein quantification was performed by bicinchoninic acid (BCA) kit (Sigma-Aldrich) following the manufacturer’s instructions. In total, 12 µg of protein were separated by sodium dodecyl sulphate-polyacrylamide gel electrophoresis (SDS-PAGE) and transferred onto PVDF membranes applying 80 V for 2 h using a wet transfer system. Then, the membranes were blocked with 5% BSA (Sigma-Aldrich) in 0.1% TBS-tween for 1 h, washed with TBS-tween and probed with a mix of rabbit anti-human antibodies (P-mTOR Ser2448, P-AKT Ser473, P-GSK-3β Ser9 and P-ERK 44/42 Thr202/Tyr204, each diluted 1:1000) overnight at 4 °C with continuous stirring. Following three 10 min washing steps, the membranes were probed with anti-rabbit or anti-mouse IgG HRP-linked antibody diluted 1:3000 for 1 h and then detected using the iBright device (Thermo Fisher Scientific, Waltham, MA, USA). After imaging, the densitometry analysis was conducted using ImageJ software version 1.53k. The total form of each evaluated protein was used as internal reference to calculate the relative phosphorylation of target proteins. Mouse anti-human β-actin was used as a loading control. All the aforementioned antibodies were purchased from Cell Signaling Technology (Danvers, MA, USA).

### 4.4. Immunocytochemistry Analysis of AKT Phosphorylation

For immunocytochemistry detection of phosphorylated AKT, cells were seeded in glass slides placed in 6-well plates at the density of 1 × 10^5^/well. Following 48 h, treatments were removed, and cells were fixed with pre-warmed 4% paraformaldehyde (PFA) (Sigma-Aldrich) for 30 min. After PFA fixation, cells were permeabilised with 0.5% Triton X-100 (Sigma-Aldrich) in PBS for 15 min. The cells were then blocked with filtered 1% milk in PBS at 37 °C for 30 min and incubated with anti-P-AKT Ser473 antibody (1:400 in 1% milk/PBS) at 37 °C for 1 h in a moisture chamber. After three washing steps with PBS, cells were incubated with fluorescent-labelled secondary antibody (from Cell Signaling Technology) (1:450 in 1% milk/PBS) at 37 °C for 1 h. Finally, images were captured using a Nikon Upright Microscope Eclipse Ci-S (Nikon, Tokyo, Japan).

### 4.5. Cell Count Assay

To evaluate the effects triggered by high insulin level on breast cancer cell proliferation, flow cytometry-based count assays were conducted using the benchtop flow cytometer MUSE (Cytek Biosciences, Fremont, CA, USA). In this regard, both the cell lines were seeded in 6-well plates at the density of 1.5 × 10^5^/well. After overnight cell adhesion in DMEM-LG with 10% FBS, the culture media was changed to the treatment media DMEM-LG or DMEM-HG with 2% FBS and with or without insulin 25 nM as previously described. After 48 h of treatment, cells were trypsinised and resuspended with DMEM-LG containing 50% FBS to inhibit trypsin. The cell suspension was diluted 20 times, mixing 20 µL of cell suspension with 380 µL of MUSE count et viability reagent (Cytek Biosciences). Following 5 min of staining incubation in the dark, cell suspensions were mixed, and cells were counted with the flow cytometer device. Representative flow cytometry plots for Cell Count Assay are provided in the supplementary section (Figure S1).

### 4.6. MTT Assay

To evaluate the effect of high-glucose containing medium or high insulin level in modulate cells proliferation and the cytotoxicity induced by the AKT inhibitor MK-2206, the MTT colorimetric assay was conducted.

To perform the MTT assays, both the cell lines were seeded in 96-well plates at the density of 1 × 10^4^/well and cultured overnight. Following adhesion, the culture media was removed from each well and changed with 150 µL of treatment media. After 48 h of treatment, cells were assayed using the MTT test (Sigma-Aldrich). An amount of 10 µL of the 5 mg/mL MTT in PBS solution was added to each well, and plates were incubated for an additional 4 h at 37 °C. The media was then removed, and formazan crystals solubilised using DMSO. The optical density (OD) was measured at 570 nm, and the background was removed by measuring the OD at 650 nM. OD was recorded using the TECAN Infinite M Plex microplate reader (Tecan Trading AG, Männedorf, Switzerland).

### 4.7. Evaluation of Cell Cycle

To evaluate the ability of high insulin level to modify the percentage of cells distributed in the three main cell cycle phases G0/G1, S, and G2/M, flow cytometer-based assays were conducted using the flow cytometer MUSE (Cytek Biosciences). Cells were seeded and treated as described for the cell count assay. After the treatments, cells were trypsinised and fixed in ice-cold 70% ethanol overnight at −20 °C. Fixed cells were washed with PBS, resuspended in 200 µL of Muse Cell Cycle Reagent (Cytek Biosciences) and incubated for 30 min at room temperature, protected from light. Following the staining, cells were mixed to obtain a uniform suspension, and the cell cycle was analysed with the flow cytometer MUSE.

### 4.8. Real-Time Cell Analysis—Migration

The xCELLigence RTCA DP Instrument (F. Hoffmann-La Roche SA, Basel, Switzerland) was used to evaluate the real-time cell migration. To evaluate the migration induced by the chemoattractant, media containing 10% FBS was dispensed in the lower-chamber wells of the CIM plates (Agilent, Santa Clara, CA, USA), while media without chemoattractant was dispensed for basal migration detection. For chemoattractant gradient formation, 50 µL of culture media without FBS was added to the upper-chamber wells and CIM plates incubated for 1 h at 37 °C. Following 48 h of cell treatment in T25 flasks, cells were trypsinised and seeded at the density of 3 × 10^4^ cells/well in the upper chamber of the CIM plates, in a volume of 100 µL of serum-free treatment media. Plates were then incubated for up to 24 h for cell migration detection.

### 4.9. Statistical Analysis

Data are expressed as mean with SEM of at least three independent experiments. Differences between two conditions were analysed by Student’s *t*-test, whereas comparisons between three or more treatments were performed using one-way ANOVA followed by Tukey’s post hoc test to correct for multiple comparisons. To compare groups on two different categorical variables, the two-way ANOVA followed by Sidak’s post hoc test were performed. A *p*-value < 0.05 was considered statistically significant. Statistical analyses were performed using GraphPad Prism for Windows version 8.0.2 (GraphPad Software, San Diego, CA, USA).

## 5. Conclusions

In conclusion, our study demonstrates that chronic high-glucose containing medium does not increase BC cell proliferation, whether alone or in combination with high insulin level. On the contrary, high insulin level only exerts distinct effects on BC cells by promoting AKT-dependent proliferation in ER-positive MCF-7 cells while enhancing the migratory potential of triple-negative MDA-MB-231 cells.

## Data Availability

The data supporting the study findings are available on request from the corresponding authors [A.P.; L.M.N.].

## Supplementary Materials

The following supporting information can be downloaded at: https://www.mdpi.com/article/10.3390/ijms26157523/s1.
