# Supplementary material for: Breast Cancer Cell Line-Specific Responses to Insulin: Effects on Proliferation and Migration
Source: Int J Mol Sci. 2025 Aug 4;26(15):7523. doi: 10.3390/ijms26157523 (PMC12347025; doi:10.3390/ijms26157523)
Supplement: Supplementary file 1 [file ijms-26-07523-s001.zip › ijms-3731198-supplementary.pdf]

A

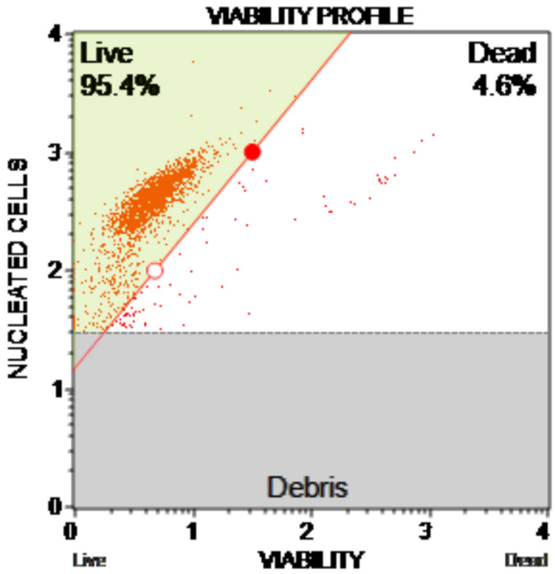

B

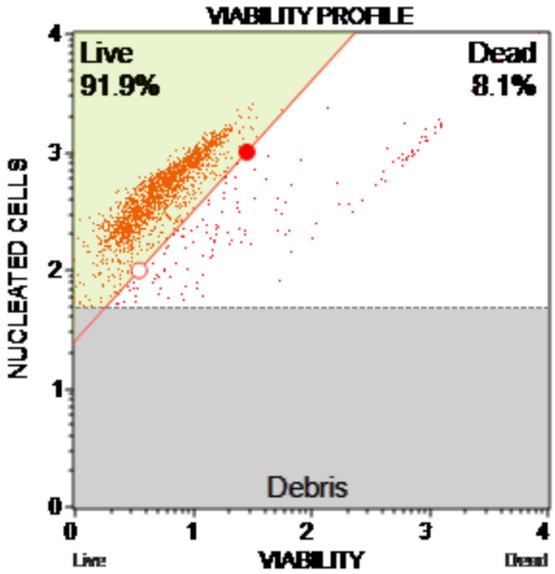

Figure S1. Flow cytometry plots of cell count assay. Representative flow cytometry plots of cell count analysis of MDA-MB-231 (**A**) and MCF-7 (**B**) cells using the MUSE system.
